# Clinical, socio-demographic, and parental correlates of early autism traits in a community cohort of toddlers

**DOI:** 10.1038/s41598-024-58907-w

**Published:** 2024-04-10

**Authors:** Oliver Gale-Grant, Andrew Chew, Shona Falconer, Lucas G. S. França, Sunniva Fenn-Moltu, Laila Hadaya, Nicholas Harper, Judit Ciarrusta, Tony Charman, Declan Murphy, Tomoki Arichi, Grainne McAlonan, Chiara Nosarti, A. David Edwards, Dafnis Batalle

**Affiliations:** 1 Department of Forensic and Neurodevelopmental Science, Institute of Psychiatry, Psychology & Neuroscience, King’s College London, 16, De Crespigny Park, London, SE5 8AF UK; 2 Centre for the Developing Brain, School of Imaging Sciences & Biomedical Engineering, King’s College London, London, UK; 3 MRC Centre for Neurodevelopmental Disorders, King’s College London, London, UK; 4 Department of Computer and Information Sciences, Faculty of Engineering and Environment, Northumbria University, Newcastle Upon Tyne, UK; 5 Department of Child and Adolescent Psychiatry, Institute of Psychiatry, Psychology & Neuroscience, King’s College London, London, UK; 6 Department of Psychology, Institute of Psychiatry, Psychology & Neuroscience, King’s College London, London, UK; 7 Department of Paediatric Neurosciences, Evelina London Children’s Hospital, Guy’s and St Thomas’ NHS Foundation Trust, London, UK; 8 Department of Bioengineering, Imperial College London, London, UK

**Keywords:** Social behaviour, Paediatric research

## Abstract

Identifying factors linked to autism traits in the general population may improve our understanding of the mechanisms underlying divergent neurodevelopment. In this study we assess whether factors increasing the likelihood of childhood autism are related to early autistic trait emergence, or if other exposures are more important. We used data from 536 toddlers from London (UK), collected at birth (gestational age at birth, sex, maternal body mass index, age, parental education, parental language, parental history of neurodevelopmental conditions) and at 18 months (parents cohabiting, measures of socio-economic deprivation, measures of maternal parenting style, and a measure of maternal depression). Autism traits were assessed using the Quantitative Checklist for Autism in Toddlers (Q-CHAT) at 18 months. A multivariable model explained 20% of Q-CHAT variance, with four individually significant variables (two measures of parenting style and two measures of socio-economic deprivation). In order to address variable collinearity we used principal component analysis, finding that a component which was positively correlated with Q-CHAT was also correlated to measures of parenting style and socio-economic deprivation. Our results show that parenting style and socio-economic deprivation correlate with the emergence of autism traits at age 18 months as measured with the Q-CHAT in a community sample.

## Introduction

Autism spectrum disorders (ASD) are typically diagnosed between 4 and 7 years of age^1,2^. The age at symptom onset however is often earlier than this, with neurodivergence first being suspected by parents in most instances between 1 and 2 years of age^3^. Autism traits, such as difficulties in social interaction and communication, and restricted behaviours and interests, are continuously distributed in the general population^4^. Screening tools aiming to quantify these traits are well established and cut-off points with high sensitivity (albeit at the cost of low specificity^5^) for predicting a future clinical autism diagnosis have been demonstrated^6,7^, although results in some real-world cohorts are less promising^8^. One such tool is the Qualitative Checklist for Autism in Toddlers (Q-CHAT)^9^. The Q-CHAT is a 25-item questionnaire with each item rated by the parents from 0 to 4. It has been validated for use in multiple countries^10–14^, and has a positive predictive value of 28% for a future ASD diagnosis (using screening at two timepoints)^15^. Autism traits exist in the population as a continuum^16^, and most individuals screened, typically developing or otherwise, will display at least some autism traits at age 18 months^17^.

The likelihood of receiving an autism diagnosis is associated with both genetic and environmental factors^18,19^, and the same may be true of early autism traits. Some factors are known to correlate with autism traits at age 18 months—for example, sex (with males scoring higher than females)^8,9,20,21^ or preterm birth^11,22^. However, beyond these factors there is a relative lack of research into what else may influence the emergence of autism traits in early life, although single studies have linked maternal nausea and vomiting during pregnancy^23^, neonatal illness^24^, maternal depression and anxiety^25,26^, immigrant mothers^27^ and lower levels of parental education^25^ with higher scores on 18-month autism screening tools. Q-CHAT score at 18 months has also been shown to be negatively correlated with general language development^11^. The broader developmental phenotype is known to be influenced by a wide range of exposures, including preterm birth^28^, neonatal illness^29^, and multiple psychosocial factors^30–33^. Given that Q-CHAT is known to correlate with general language development, it is reasonable to hypothesise that Q-CHAT scores may themselves be influenced by these same exposures.

As well as research using structured tools there are previous studies which examine exposures associated with single features of social communication development in toddlerhood. Multiple factors including less responsive or less effective maternal parenting styles^34,35^, greater maternal depression and experience of trauma^36^ and a lower quality home environment^37^ have been correlated with less favourable social communication development in toddlerhood.

Because greater autism trait emergence at age 18 months is associated with a greater likelihood of childhood autism^15^ understanding correlates of the Q-CHAT score at 18 months may help us to understand what early life experiences are associated with an increased likelihood of a future autism diagnosis in some individuals. The developing Human Connectome Project (dHCP) has collected Q-CHAT scores, other neurodevelopmental measures and demographic information from a large cohort of 18-month-old toddlers in London, UK. Using this dataset, we aimed to characterise correlates of Q-CHAT score. We hypothesised that, in keeping with the known associations between early life adversity and other measures of neurodevelopment, we would observe a pattern of psychosocial adversity being associated with higher Q-CHAT scores. Relationships between variables and Q-CHAT score are presented in both univariable (in part to inform future studies which may only have some of our variables available) and multivariable models. We use models with scores from the Bayley Scales of Infant and Toddler Development, 3rd Edition (BSID-III)^38^ additionally included as covariates in order to understand whether any relationships between early life experiences and autism traits are influenced by general neurodevelopment.

## Methods

### Sample

This study is based on a sample of neonates participating in the Developing Human Connectome Project (dHCP, http://www.developingconnectome.org/). Participants were all recruited at St Thomas’ Hospital, London, UK. There were no specific inclusion or exclusion criteria for enrolment in this study, and recruitment was primarily from the antenatal clinic with no specific stratification.

Toddlers were invited for neurodevelopmental assessment at 18 months post-expected delivery date; appointments were made according to family availability as close as possible to this time-point. The only inclusion criterion for this manuscript from the overall cohort was completion of the neurodevelopmental assessment. There were no exclusion criteria.

The dHCP received United Kingdom National Health Service research ethics committee approval (14/LO/1169, IRAS 138070), and was conducted in accordance with the World Medical Association’s Code of Ethics (Declaration of Helsinki). Written informed consent was obtained from parents at recruitment into the study.

### Data collection

Data collection took place either at St Thomas’ Hospital, London, UK, or via questionnaires distributed to the participants’ parents. At the time of birth, clinical variables, gestational age at birth and sex were extracted from the medical records of participants in the study; and maternal age, maternal pre-pregnancy BMI, and parent Autism/Attention Deficit Hyperactivity Disorder (ADHD) history were also collected via a maternal questionnaire. The last of these was asked in the format “Have you or your child’s biological father ever been diagnosed with Attention Deficit Hyperactivity Disorder (ADHD) or Autism?” This was a yes/no question.

At the time of birth, the socio-demographic status of participant families was recorded as measured by the Index of Multiple Deprivation Rank (IMD), a postcode-based score assigned to every address in the UK which gives a composite measure of socio-economic disadvantage, based on the mother’s address at the time of birth. A lower score corresponds to greater geographical deprivation, with 1 being the lowest score possible (most deprived) and 32,844 being the highest score possible (least deprived). The Index is itself drawn from 39 sub-scales, grouped into 7 categories of deprivation: income, employment, health deprivation and disability, education, skills and training, crime, barriers to housing and services, and living environment^39^.

Further socio-demographic information was collected by questionnaire: maternal age at leaving education (“At what age were you last in full time education?”), maternal first language (“Is English your first language”?), and parent cohabiting status. The Cognitively Stimulating Parenting Scale (CSPS), a questionnaire assessing the availability of resources to support cognitively stimulation parenting, associated to both parenting style and socio-economic deprivation was also collected^40,41^. The CSPS was updated to include items relating to access to mobile phones and apps. A higher score is indicative of a more stimulating home environment, with a minimum possible score of 0 and a maximum possible score of 40. The Q-CHAT score (a parent reported questionnaire) was collected at the time of 18-month follow-up. This gives a score between 0 and 100, with higher scores indicative of more autism traits. The Bayley Scales of Infant and Toddler Development, 3rd edition (BSID-III)^38^, was administered by either a Chartered Psychologist or Paediatrician when the children were 18 months of age. The BSID-III Cognitive, Motor, and Language composite scores were used for analysis in this study. Two measures of parenting style were also collected at this time. The first of these, the Parenting Scale^42^, is a self-reported tool that measures three different dimensions of parenting: Laxness, the tendency to behave passively and give in to misbehaviour; Over-reactivity, which measures anger, meanness and irritability in parenting; and Verbosity, a measure of parental dependence on talking even when ineffective as a discipline style. The dimensions have a minimum score of 1, and a maximum of 7. The Edinburgh Postnatal Depression Scale (EPDS) was also completed at follow-up. This is a well-established self-reported tool for quantifying postnatal depressive symptoms, with a minimum of 0 and a maximum of 30. Higher ranks are indicative of more depressive symptoms^43^.

### Statistical analysis

Univariable associations between variables and Q-CHAT score were tested by Pearson’s correlation or t-test as appropriate. Multivariable associations between variables of interest and Q-CHAT score were assessed by generalized linear model (GLM). Statistical significance was tested with random permutation tests, using 10,000 permutations. P-values are reported uncorrected, with those surviving multiple comparisons via false discovery rate (FDR) indicated^44^. Principal component analysis was used to characterize the latent structure of independent variables, and to address collinearity between linear variables. The “elbow method”^45^ was used to determine the optimal number of principal components (PCs) to use in later analyses. Associations between PC scores and the original input variables was determined by Pearson’s correlation, with p < 0.05 after FDR correction considered significant.

Analyses were performed and figures made using Rstudio v4.0.2 (Rstudio, MA, U.S.A). The “FDRestimation”, and “corrplot” packages were additionally used^46,47^. PCA was performed using the “prcomp” function from base R rather than a dedicated package. Our code to implement random permutation tests for GLMs in R is available at: https://github.com/CoDe-Neuro/ptestR.

## Results

### Population

At the time of the study commencing, 644 individuals in the dHCP dataset had a Q-CHAT score available. Of these 536 had a complete set of demographic data and were included in the study. A comparison between individuals included and excluded is shown in Supplementary Table S1. There were some differences between those included and excluded—individuals included in the study experienced on average lower geographical deprivation (higher IMD Rank), lower maternal depression, and less extreme parenting styles. The characteristics of the sample used, and the univariate relationships of each variable to Q-CHAT score are shown in Table 1, the distribution of Q-CHAT scores is shown in Supplementary Fig. S1.

**Table 1:** Sample characteristics.

**Outcome variable**		
Total Q-CHAT score, mean (SD), range	30.1 (5.9), 8–70	–
**Clinical variables**		**r (p)**
Age at follow-up [months], mean (SD), range	18.8 (1.6), 16–26	0.010 (0.535)
Gestational age at birth [weeks], mean (SD), range	38.1 (3.9), 23.0–43.0	− 0.067 (0.120)
BMI [kg/m^2^], mean (SD), range	24.2 (4.4), 15.3–43.4	**0.093 (0.030)**
Mother age [years], mean (SD), range	34.3 (4.7), 17–52	**− 0.105 (0.014)**
		**t (p)**
Sex [male (0), female (1)], N (%)	278 (52%), 258 (48%)	1.820 (0.068)
Parent ASD/ADHD diagnosis [yes (1), no (0)], N (%)	28 (5%), 508 (95%)	− 0.4246 (0.674)
Socio-demographic variables		**r (p)**
IMD rank, mean (SD), range	14,626.2 (7409.2), 2410–32,726	− 0.190 (< 0.001)*
CSPS, mean (SD), range	20.5 (3.5), 7–28	− 0.219 (< 0.001)*
Mother education [years], mean (SD), range	23.6 (4.5), 12–43	0.001 (0.957)
		**t (p)**
Mother 1st language [English (1), other (0)], N (%)	338 (63%), 198 (37%)	4.518 (< 0.001)*
Parents cohabiting [yes (0), no (1)], N (%)	520 (97%), 16 (3%)	− 1.650 (0.119)
Parental-psychological variables		**r (p)**
Mother laxness, mean (SD), range	2.9 (0.8), 1–5.6	0.286 (< 0.001)*
Mother over-reactivity, mean (SD), range	2.2 (0.7), 1–5.1	0.180 (< 0.001)*
Mother verbosity, mean (SD), range	3.4 (0.8), 1–6.4	0.300 (< 0.001)*
Mother EPDS, mean (SD), range	4.5 (4.2), 0–28	0.127 (< 0.001)*
BSID-III cognitive composite, mean (SD), range	101.0 (11.1), 55–130	− 0.358 (< 0.001)*
BSID-III language composite, mean (SD), range	98.2 (15.4), 47–153	− 0.528 (< 0.001)*
BSID-III motor composite, mean (SD), range	101.5 (10.2), 52–130	− 0.267 (< 0.001)*

Mean, standard deviation, and range displayed for linear variables. Frequency displayed for categorical variables. Correlations to QCHAT calculated by Pearson’s r or t-test as appropriate. Significant univariable correlations are shown in bold.

*Significant after FDR multiple comparison correction (α< 0.05).

Five variables were positively correlated with Q-CHAT score: BMI (r = 0.093, p = 0.030), EPDS (r = 0.127, p < 0.001), and three measures of maternal parenting style, laxness (r = 0.286, p < 0.001), over-reactivity (r = 0.180, p < 0.001), and verbosity (r = 0.300, p < 0.001). Mother’s age (r = − 0.105, p = 0.014), IMD rank (r = − 0.190, p < 0.001) and CSPS score (r = − 0.219, p < 0.001) were negatively correlated with Q-CHAT score. The correlations with BMI and mother’s age did not survive FDR however. Total Q-CHAT scores were significantly higher in individuals whose mothers spoke a language other than English as their first language (t = 4.52, p < 0.001). All BSID-III composite scores were negatively associated with Q-CHAT score. The strongest association was with Language Composite Score (r = − 0.528, p < 0.001).

### Multivariable models of Q-CHAT score

We assessed the association of all variables with Q-CHAT score in two separate multivariable models, with or without the addition of BSID-III composite scores to control for the effect of general neurodevelopment, identifying specific relationships between demographic variables and Q-CHAT score (Table 2).

**Table 2:** General linear model of Q-CHAT with clinical, socio-demographic, and parental variables with or without the addition of BSID-III Cognitive, Motor and Language Composite Scores to the model.

	Without BSID-III		With BSID-III	
	r^2^ (Adj. r^2^) = 0.20(0.19), p < 0.001		r^2^ (Adj. r^2^) = 0.36(0.34), p < 0.001	
	t	p	t	p
Gestational age at birth	− 1.94	0.052	− 0.45	0.646
BMI	0.57	0.567	0.95	0.341
Mother age	− 0.73	0.461	− 1.17	0.239
Sex	**− 2.32**	**0.020**	− 0.83	0.407
Parent ASD/ADHD diagnosis	0.57	0.567	− 0.23	0.813
IMD rank	**− 2.56**	0.010*	**− 2.06**	**0.039**
CSPS	**− 3.38**	< 0.001*	− 1.17	0.238
Mother education	− 0.08	0.929	0.35	0.719
Mother 1st language English	− 1.58	0.113	− 0.01	0.994
Parents cohabiting	1.74	0.082	1.49	0.135
Mother laxness	**3.79**	< 0.001*	**2.68**	0.007*
Mother overreactivity	1.11	0.269	1.04	0.297
Mother verbosity	**3.29**	0.001*	**3.39**	< 0.001*
Mother EPDS	1.72	0.080	1.26	0.206
Cognitive composite	NA	NA	− 0.28	0.779
Language composite	NA	NA	**− 8.32**	< 0.001*
Motor composite	NA	NA	− 0.41	0.677

Non-reference categories are as follows: Sex—Male, Parent ASD/ADHD Diagnosis—Yes, mother 1st language—not english. Bold indicates p < 0.05, * indicates significance after FDR correction.

*Significant after FDR multiple comparison correction (α < 0.05).

A multivariable model without BSID-III explained 20% of Q-CHAT variance. After FDR correction four variables were individually associated with Q-CHAT score: IMD Rank (t = − 2.56, p = 0.010) and CSPS (t = − 3.38, p < 0.001) were negatively associated and Mother Laxness (t = 3.79, p < 0.001) and Mother Verbosity (t = 3.29, p = 0.001) were positively associated. After adding BSID-III composite scores to the model, two of these (Mother Laxness and Mother Verbosity) remained significantly associated with Q-CHAT score (t = 2.68, p = 0.007 and t = 3.39, p < 0.001 respectively), in addition to BSID-III language composite score (t = − 8.32, p < 0.001), which was negatively associated with Total Q-CHAT score. Notably sex and parent ASD/ADHD diagnosis status did not correlate individually with Q-CHAT score in either model after FDR multiple comparison correction.

**Figure 1:**
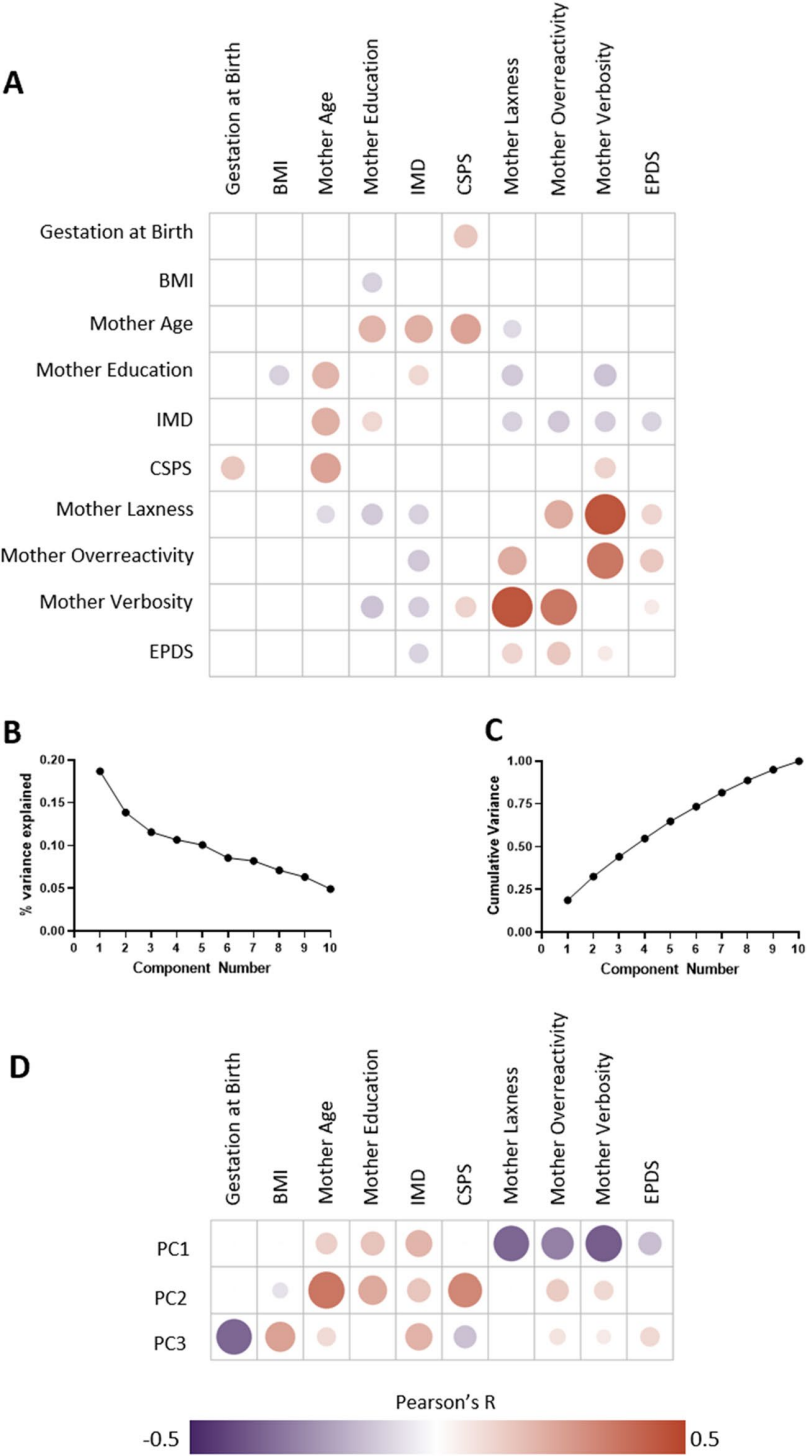
Principal Component Analysis of linear variables. (**A**) Correlogram of associations between linear variables. Pearson’s r indicated for correlations with p < 0.05. (**B**) Scree plot of PCA components (**C**) Cumulative variance plot of PCA components (**D**) Correlations of original linear variables to principal components. Correlation indicated by size and colour of circle. Only correlations remaining significant (p < 0.05) after FDR correction are shown. Values of each correlation are shown in Supplementary Table S2, and variable weights in Supplementary Table S3.

A limitation of interpreting these models is the collinearity between demographic variables (Fig. 1A). In order to address this issue without removing variables from the model, we performed a PCA of the linear variables to obtain orthogonal components, which we then used in a general linear model in place of the original linear variables^48^. We selected the first 3 principal components (PCs) to represent our data (Fig. 1B,C), which explained 19%, 14% and 12% of variance respectively. The multivariable models associating demographic variables and BSID-III composite scores with Q-CHAT score were subsequently repeated, with linear variables being replaced by PCA components 1–3 (Table 3). Details of variable correlations with each PC are shown in Fig. 1D. PC1 captures lower maternal laxness, overreactivity and verbosity, and lower socio-economic deprivation (higher IMD rank); PC2 is associated with higher maternal age, greater maternal education, lower socio-economic deprivation (higher IMD rank) and a more stimulating home environment (higher CSPS); and PC3 is associated with clinical adversity (lower gestational age at birth, higher maternal BMI and maternal EPDS).

**Table 3:** General linear model of the association between demographic variables, BSID-III composite scores and Q-CHAT.

	Without BSID-III		With BSID-III	
	r^2^ (Adj. r^2^) = 0.17(0.16), p < 0.001		r^2^ (Adj. r^2^) = 0.35(0.33), p < 0.001	
	t	p	t	p
PC1	**− 8.17**	< 0.001*	**− 6.59**	< 0.001*
PC2	− 1.86	0.062	− 0.70	0.480
PC3	1.29	0.194	0.10	0.913
Sex	**− 2.02**	**0.043**	− 0.48	0.630
Parent ASD/ADHD diagnosis	0.59	0.550	− 0.26	0.790
Mother 1st language English	− 1.86	0.063	− 0.06	0.948
Parents cohabiting	**2.04**	**0.041**	1.51	0.129
Cognitive composite	NA	NA	− 0.55	0.577
Language composite	NA	NA	**− 8.96**	< 0.001*
Motor Composite	NA	NA	− 0.15	0.880

Linear variables were first transformed into orthogonal components via PCA. PC1 captures variable associations which are associated with less expressive parenting styles and low socio-economic deprivation, PC2 is associated with higher maternal age, and a more stimulating home environment, and PC3 is associated with variables describing clinical adversity.

Bold indicates p < 0.05, * indicates significance after FDR multiple comparison correction (α < 0.05).

Approximately 17% of Q-CHAT variance was explained by a model including 3 PCs and the categorical variables only, with only PC1 remaining statistically significant in the model after FDR correction (t = − 8.17, p < 0.001, Table 3). Approximately 36% of Q-CHAT variance was explained by the model when including BSID-III scores to account for general neurodevelopment, with both PC1 and BSID-III language composite scores statistically significant (t = − 6.59 and t = − 8.96 respectively, p < 0.001, Table 3).

PC1 (associated with lower maternal laxness, overreactivity, verbosity scores and lower socio-economic deprivation) was negatively correlated with Q-CHAT score (t = − 8.17, p < 0.001)—i.e., individuals experiencing a lax, overreactive and verbose parenting style, and high socio-economic deprivation (low IMD), have higher Q-CHAT scores (more autism traits). PC1 is also positively correlated with maternal age, maternal age at last full-time education, and negatively correlated with EPDS. It is worth noting that sex and parent ASD/ADHD diagnosis status did not correlate with Q-CHAT score in either model.

## Discussion

We observed correlations of Q-CHAT score with measures of parenting style and measures of socio-demographic adversity, with the former category demonstrating the strongest associations. Conversely, some variables known to increase the likelihood of an autism diagnosis in later childhood, such as male sex^49^, a family history of autism^50^ and gestational age at birth^51^ were not associated with Q-CHAT scores.

A multivariable model of demographic variables explained 20% of Q-CHAT variance. In this model four variables (two measures of socio-economic deprivation and two measures of parenting style) were individually significantly associated with Q-CHAT score. Adding measures of general neurodevelopment to this model increased the explained variance to 36%, however this also resulted in two variables, IMD Rank and CSPS (measures of socio-economic deprivation) no longer being individually significantly associated with Q-CHAT score. Taken together this suggests that maternal parenting style is specifically associated with Q-CHAT score, whereas that the association of socio-economic deprivation with Q-CHAT is partially explained by general neurodevelopment.

Maternal verbosity had the strongest association with Q-CHAT score of any variable tested, remaining significantly associated with Q-CHAT score in multivariable models with and without general neurodevelopment. The mechanism via which this association occurs is unknown, but several pathways are plausible. Parenting and affection display styles are heritable traits, and it may be that the genetic and environmental factors contributing to adverse parenting styles also contribute to autism trait emergence in toddlerhood^52,53^. Previous studies have suggested that parenting styles directly influence childhood behaviour, as children learn by repetition^54,55^. Parent–child relationships of children with childhood autism diagnoses are also more likely to be discordant than those of neurotypical offspring^56^. This discordance is thought to be both a cause and consequence of difficulties in social understanding^57,58^, and it is possible that even at 18 months toddlers displaying more autism traits have greater difficulty relating to their parents, leading to greater discordance^59,60^. In support of this hypothesis a recent randomised controlled trial demonstrated that a 10-session therapist delivered parenting skills intervention, which promoted concordant interaction, led to a roughly threefold reduction in autism diagnoses 2 years later^61^. However, parenting styles are at least partly heritable^62^, hence it is also possible that the offspring of parents who naturally display more verbose and less collaborative parenting styles experience more difficulties developing social relationship abilities, and thus score more highly on the Q-CHAT. A final possibility is that maternal verbosity is in part a proxy measure of other forms of adversity: Verbosity has been previously shown to correlate with multiple measures of maternal stress^63^, which in turn has been reported to correlate with a higher likelihood of offspring autism^64^. All dimensions of parenting style are correlated with IMD rank in our data (Fig. 1A), and this is in keeping with a body of literature demonstrating associations between parenting style and socio-economic status^65^. A more deeply phenotyped sample would be required to investigate how and if these different factors influence the relationship between maternal verbosity and Q-CHAT score. We do not seek to suggest that the emergence of autism traits is something parents can control, and a final possible interpretation of the correlation between maternal parenting style and autism trait emergence is reporting bias. Given that both the Q-CHAT and the parenting style questionnaire are self-reported tools, individual patterns of response could relate to a wide number of factors, including mental state, intellectual ability, and neurodevelopmental profile. Future studies could consider clinician administered measures to address this issue. There are limitations to our findings on parenting style. Firstly, we did not ask any questions about family composition or care arrangements beyond parent cohabiting status – we therefore do not know if the mother was the primary caregiver for each child included. Secondly, we should note that the Parenting Scale captures only some dimensions of parenting. Other dimensions, including many usually thought of as positive, are correlated with offspring temperament development, and are not considered in our data^66^. As a related point it is not currently well understood how the dimensions of the Parenting Scale correlate to other parenting style constructs which may affect early neurodevelopment^67^, and it may be the case that the apparent correlation here between Verbosity and Q-CHAT is in fact mediated by a hidden factor.

Based on previous literature, some of our results are expected, while others are unexpected. For instance, we showed that multiple measures of psychosocial disadvantage correlate with higher Q-CHAT scores. There is a significant body of evidence demonstrating that early life adversity affects several domains of early childhood behaviour, including cognitive^30^, motor^31^, and language^68^ development, as well as emerging psychopathologies^26,69^. It is known that lower socio-economic status correlates with higher scores on the precursor to the Q-CHAT, the M-CHAT^33^. Also, one previous study has specifically reported higher Q-CHAT scores in the offspring of depressed mothers^25^. Therefore, our finding that maternal depressive symptom burden, measured using EPDS, correlates with offspring Q-CHAT score is not unexpected. Our finding that two measures of social adversity correlate with higher Q-CHAT score is in keeping with existing knowledge about neurodevelopment: univariable association between maternal first language and Q-CHAT score is also in keeping with a body of previous literature which demonstrates a higher rate of autism diagnoses in children from immigrant backgrounds^70^. It is likely that parent first language not being English in our sample represents an increased risk of experiencing other adversities^71^, rather than inferring that being raised in a bilingual environment has an effect on autism trait emergence, which is not thought to be the case^72^.

We unexpectedly found no association between sex and Q-CHAT score in any analysis performed. A handful of previous studies have demonstrated higher Q-CHAT scores in male toddlers compared to female toddlers, with small but significant average score differences (3.1^73^, 3.1^74^ and 1.9^9^) reported. It is not immediately obvious why we do not see the same difference in our data, although it may be that in a larger sample this difference would have been apparent. Males in our cohort did in fact score 1.4 Q-CHAT points higher than females on average (Cohen’s d = 0.16), but the difference is not statistically significant. Similarly in a multivariable model the individual correlation between sex and Q-CHAT score is apparent (t = − 2.32, p = 0.020, Table 2) but did not survive FDR correction. It would be more appropriate to say that we could not conclude that males had higher Q-CHAT scores in our data than that there is no association at all.

We also found no significant association between parental history of ASD and Q-CHAT score in any analysis performed. A difference may reasonably have been expected based on the known familial increased likelihood of autism and ADHD diagnoses^50,75^. To date, one study has directly reported on the association between parental history of ASD and Q-CHAT score and found a large group difference, with the familial ASD history group having higher Q-CHAT scores at age 16–30 months^76^. One other study has specifically examined the difference between Q-CHAT scores in individuals with and without an older sibling with autism, and also found significant group differences^77^. It is not clear why we do not see the same effect here, although there are several possibilities. It is possible that the method in which we recorded family history was too narrow: the carer completing the questionnaire was asked only if they or their partner had ever been diagnosed with autism or ADHD, where a more broad dimensional assessment would have been preferrable. It is also possible that considering ASD and ADHD together has added noise to our data. ASD is considered to have shared aetiology with autism trait emergence^4^, but the same correlation has not been demonstrated for ADHD and autism trait emergence. Alternatively or additionally, it may be the case that we lacked sufficient positive cases (28 parents reported an ASD or ADHD diagnosis compared to 506 with no diagnosis) to have determinative power. Parents were also asked if the child participating in the study had an older sibling with an autism or ADHD diagnosis—as only 206 individuals had older siblings, we have not included this variable in the main analysis. There was similarly no difference (t = − 0.51, p = 0.62) in mean Q-CHAT score between those with (n = 23, mean Q-CHAT = 31.4) and without (n = 183, mean Q-CHAT = 30.1) an older sibling with a neurodevelopmental diagnosis. This may again be due to an insufficient number of positive cases for determinant power.

It has been previously reported that preterm birth confers an increasing likelihood of both childhood autism diagnosis and greater early autism trait emergence^78,79^. One previous study reports Q-CHAT scores in a cohort of toddlers born before 30 weeks of gestation, who scored a mean of 33.7^11^, although to our knowledge no direct comparison of Q-CHAT scores in individuals born term and preterm has yet been presented. In our cohort we find no association between gestational age at birth and Q-CHAT score directly through univariable or multivariable associations, or indirectly via PCA latent components. One possibility is that early life autism trait emergence is less readily detected by screening tools in some preterm children^80,81^. Although we have used gestational age as a linear variable, if we consider preterm birth as a binary variable there is also no difference between groups. The mean Q-CHAT scores in individuals born before 30 weeks gestation in our sample (n = 36) is however 34.6, which is in keeping with the 33.7 average score reported by Wong et al.^11^ using the same criteria. Finally, it is worth noting that inter-individual differences in the degree of immediate morbidity following preterm birth are of particular importance in later autism trait emergence^82^, variables relating to which we do not have in our models.

A finding of particular interest is how associations between demographic variables and Q-CHAT score were influenced by general neurodevelopment, which in our study we measure with BSID-III scores. All BSID-III composite scores correlated individually to the Q-CHAT score (Table 1). In a multivariable model without BSID-III scores, four variables (two socio-demographic measures, and two measures of parenting style) were significantly associated with Q-CHAT score (Table 2). With BSID-III composite scores added to the Q-CHAT model, the two socio-demographic associations were no longer significant, although the BSID-III language composite score association was. This is possibly in part due to co-linearity of the input variables (Fig. 1A). After transforming linear variables into latent orthogonal components with PCA, PC1 (associated with lower maternal laxness, overreactivity, verbosity, and lower socio-economic deprivation), was negatively associated with Q-CHAT score with or without BSID-III variables included as a confounders in the model—i.e., early life adversity was associated with more autism traits (Table 3). PC1 was significantly associated with Q-CHAT score in models with and without BSID-III scores included, suggesting that socio-demographic and parental factors are specifically influencing autism trait development as opposed to solely having a general effect on neurodevelopment. Using PC1’s correlations with the original variables, we can see how they contributed to Q-CHAT score (Fig. 1D). Some of the variables contributing to PC1 are expected, based on our univariable results and previous literature; via PC1, greater maternal laxness, overreactivity, verbosity, maternal depression, and socio-economic deprivation are associated with more autism traits. Two variables however correlate in a less intuitive fashion. Firstly, via PC1, maternal age is negatively correlated with Q-CHAT score—meaning that the offspring of older mothers have fewer autism traits (Fig. 1D). This is not in keeping with a significant body of literature that suggests that the offspring of older parents have a higher likelihood of autism^83^. One possible explanation is that there are aspects of socio-economic deprivation that we are not capturing with our variables, for example income or wider availability of family support, which may be related to both parental age and autism trait development. Secondly, maternal age at leaving full time education is negatively correlated to Q-CHAT score via PC1, suggesting maternal education is negatively correlated with autism traits at 18 months in our data. This is not in keeping with the one previous exploratory study to report on this association^25^. There is a larger body of work regarding associations of parental education and childhood autism diagnoses, with some research suggesting that autism is more commonly diagnosed in the offspring of highly educated parents^84^. Previous studies have suggested a variety of possible mechanisms, including greater access to medical professionals in more affluent families^85^, diagnostic overshadowing^86^, and stigmatising views towards autism sometimes held by less educated parents^87^. These mechanisms may not apply when investigating traits in a community sample, which may explain why we find education to be associated with a lower Q-CHAT score.

There are some limitations to our findings. The cohort used is from a single study centre, and therefore may not be representative of the wider population within the UK. It is also unknown how our results would translate to other cultures—awareness of autism as a concept varies in different geographies^88^, and awareness is linked to the perceived presence of early life traits^89^. The sub-sample included in this study also differs from those excluded, in general experiencing less psycho-social adversity, with differences observed in IMD Rank, maternal parenting style and EPDS score. The nature of the scale is itself also a limitation: the Q-CHAT is parent rated, and therefore is indicative of the parent’s subjective assessment of their child, rather than an objective test^24^; it is thus possible that reporting bias with common method variance could have altered our results. Finally, whilst studying early autism trait emergence may help us to understand typical and atypical development, it is important to be clear that autism traits measured at a single timepoint are not necessarily correlated to the likelihood of a later autism diagnosis.

A general linear model of all socio-demographic factors studied explained 20% of the variance of Q-CHAT score. Whilst this is a promising finding there are clearly a number of non-studied factors which may contribute to individual patterns of autism trait emergence, including genetics and medical comorbidities. Although emerging traits at age 18 months increase the likelihood of a future diagnosis of autism, the positive predictive value of a high Q-CHAT score (or indeed a high score on any early autism screening tool) is low^90^. The prevalence of childhood autism in the UK is approximately 1.8%^91^. If this prevalence is seen in our cohort then approximately 10 individuals may be expected to receive an autism diagnosis, meaning that what we are largely studying here are variations in the spectrum of typical development, which may^92^ or may not^93^ be of any real world relevance. Some of our more unexpected findings (for example the lack of a robust association between Q-CHAT score and sex) may in part be explained by a difference between the underlying nature of a clinical autism diagnoses and the expression of autism traits in the wider population. We hope in future to follow-up this cohort in childhood, which will allow us to re-analyse if the same factors we find here to be predictive of autism trait emergence are also predictive of diagnostic status.

Our findings suggest some possible avenues for future research. Deeply phenotyped and well powered prospective cohort studies of childhood autism are needed but given the prevalence of the condition sample sizes would need to be extremely large to allow for firm conclusions to be drawn. A more logistically favourable approach to further examining some of the antecedents of autism trait development that we (and other authors) have proposed would be to focus on groups hypothesised to be more likely to develop a high level of traits. This study design is well established when examining the sequelae of a family history of autism^94^, and has also been used to study the effects of parental immigration^71^ and depression^95^. We suggest that a cohort experiencing severe psycho-social deprivation, and optimally screened for early autism traits prior to being followed up in later childhood to confirm diagnostic status, is a potential avenue in the study of early life autism traits.

## Conclusions

Autism traits at age 18 months in a community sample are associated with several prior exposures, most significantly parenting styles. In multivariable models 20% of variance of Q-CHAT score can be explained by socio-economic and parental factors, with the universal finding being that a less favourable environment results in a higher Q-CHAT score (more autism traits). Our results are of potential interest from two perspectives. Firstly, future authors investigating the Q-CHAT score and other measures of early autism traits should be aware of our findings as potential confounders or limiting factors in their work. Secondly, in our study we find less well studied correlates of autism trait development (such as parenting style and social deprivation) to be more influential than sex and family history—are our results unique, or do we need to consider a broader range of factors when discussing autism trait emergence?

### Supplementary Information

Supplementary Information.

## Data Availability

The dHCP is an open-access project. Data from the project can be downloaded by registering at https://data.developingconnectome.org/app/template/Login.vm. Analyses presented here include data to be included in future releases.
