# Supplementary material for: Clinical, socio-demographic, and parental correlates of early autism traits in a community cohort of toddlers
Source: Sci Rep. 2024 Apr 10;14:8393. doi: 10.1038/s41598-024-58907-w (PMC11006842; doi:10.1038/s41598-024-58907-w)
Supplement: Supplementary file 1 — Supplementary Information. [file 41598_2024_58907_MOESM1_ESM.pdf]

Supplementary Material

Supplementary Table S1 – *Comparison of individuals included (n=536) and excluded (n=108) from analysis. Group differences assessed by t-test or chi-squared test as appropriate.*

	**Mean Included**	**Mean Excluded**	**t**	**p**
Total Q-CHAT Score	30.1	31.2	1.00	0.318
Gestational Age at Birth [Weeks]	38.2	38.9	1.74	0.083
BMI	24.7	24.1	0.96	0.334
Mother Age [Years]	33.9	34.3	-0.88	0.377
	**M/F Included**	**M/F Excluded**	**χ^2**	**p**
Sex	278/258	65/43	2.17	0.140
	**Y/N Included**	**Y/N Excluded**		
Parent ASD/ADHD Diagnosis	28/508	5/96	0.13	0.990
	**Mean Included**	**Mean Excluded**	**t**	**p**
IMD Rank	14588	11712	**-3.96**	**<0.001**
CSPS	20.5	20.5	-0.08	0.932
Mother Education	23.2	23.6	-0.86	0.388
	**Y/N Included**	**Y/N Excluded**	**χ^2**	**P**
Mother 1st Language English	338/198	55/49	3.38	0.065
	**Y/N Included**	**Y/N Excluded**		
Parents Cohabiting	520/16	103/15	0.337	0.561
	**Mean Included**	**Mean Excluded**	**t**	**P**
Mother Laxness	2.9	3.2	**2.71**	**0.007**
Mother Overreactivity	2.2	2.3	1.46	0.142
Mother Verbosity	3.4	3.6	**2.25**	**0.025**
Mother EPDS	4.5	2.6	**-3.31**	**0.001**

Supplementary Table S2 *– Correlates between PC1-3 and linear variables. PC1 is associated with positive parenting styles and low socioeconomic deprivation, PC2 is associated with low socioeconomic deprivation with expressive parenting styles, and PC3 is associated with clinical adversity.*

	**PC1**		**PC2**		**PC3**	
	**r**	**p**	**r**	**p**	**r**	**p**
Gestational Age at Birth	-0.07	0.083	0.09	0.304	**-0.69**	**<0.001***
BMI	-0.01	0.886	**-0.13**	**0.017***	**0.49**	**<0.001***
Mother Age	**0.25**	**<0.001***	**0.72**	**<0.001***	**0.19**	**<0.001***
IMD Rank	**0.31**	**<0.001***	**0.45**	**<0.001***	-0.01	0.783
CSPS	**0.39**	**<0.001***	**0.30**	**<0.001***	**0.40**	**<0.001***
Mother Education	-0.08	0.427	**0.63**	**<0.001***	**-0.28**	**<0.001***
Mother Laxness	**-0.69**	**<0.001***	**0.10**	**0.019***	0.07	0.084
Mother Overreactivity	**-0.57**	**<0.001***	**0.27**	**<0.001***	**0.14**	**0.001***
Mother Verbosity	**-0.73**	**<0.001***	**0.20**	**<0.001***	**0.11**	**0.008***
Mother EPDS	**-0.29**	**<0.001***	0.01	0.819	**0.20**	**<0.001***

**p value remains significant after FDR multiple comparison correction*

Supplementary Table S3 *– Variable weights of PC1-3*

	**PC1**	**PC2**	**PC3**
Gestational Age at Birth	-0.077	0.07	-0.66
BMI	0.01	-0.11	0.47
Mother Age	0.19	0.61	0.18
IMD Rank	-0.07	0.54	-0.27
CSPS	0.23	0.38	-0.02
Mother Education	0.30	0.25	0.36
Mother Laxness	-0.51	0.08	0.09
Mother Overreactivity	-0.43	0.23	0.11
Mother Verbosity	-0.55	0.17	0.13
Mother EPDS	-0.21	-0.01	0.21

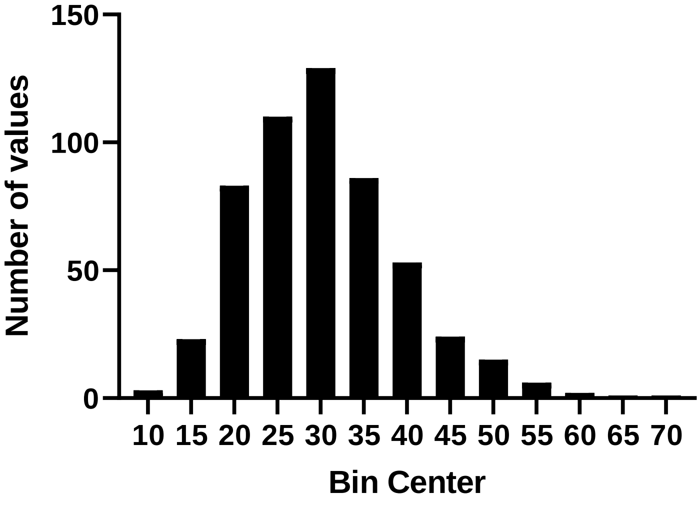

Supplementary Figure S1 – *Frequency Distribution of Q-CHAT scores. Shapiro Wilk W=0.973, p<0.001. 90/536 individuals scored 39 (the cut-off proposed for further investigation by Allison et al, 2021) or greater.*
